# Pictures of Sir M. McKenzie and Pasteur

**Published:** 1889-01

**Authors:** 


PICTURES OF SIR MORELL MCKENZIE AND M. LOUIS PASTEUR.—We thank Messrs. Parke, Davis & Co. for the beautiful large size portraits of the above distinguished gentlemen, the one a specialist in throat affections, made famous as the English Consulting Physician to the late Emperor of Germany; the other, the great French Master in Bacteriology.

